# People judge others more harshly after talking to bots

**DOI:** 10.1093/pnasnexus/pgae397

**Published:** 2024-09-19

**Authors:** Kian Siong Tey, Asaf Mazar, Geoff Tomaino, Angela L Duckworth, Lyle H Ungar

**Affiliations:** Department of Management and Strategy, University of Hong Kong, Hong Kong, Hong Kong; Wharton School of Business, University of Pennsylvania, Philadelphia, PA 19104, USA; Marketing Department, University of Florida, Gainesville, FL 32611, USA; Department of Psychology and Wharton School of Business, University of Pennsylvania, Philadelphia, PA 19104, USA; Computer and Information Science Department, University of Pennsylvania, Philadelphia, PA 19104, USA

**Keywords:** artificial intelligence, human–AI interaction, spillover, judgment

## Abstract

People now commonly interact with Artificial Intelligence (AI) agents. How do these interactions shape how humans perceive each other? In two preregistered studies (total *N* = 1,261), we show that people evaluate other humans more harshly after interacting with an AI (compared with an unrelated purported human). In Study 1, participants who worked on a creative task with AIs (versus purported humans) subsequently rated another purported human's work more negatively. Study 2 replicated this effect and demonstrated that the results hold even when participants believed their evaluation would not be shared with the purported human. Exploratory analyses of participants’ conversations show that prior to their human evaluations they were more demanding, more instrumental and displayed less positive affect towards AIs (versus purported humans). These findings point to a potentially worrisome side effect of the exponential rise in human–AI interactions.

## Introduction

People now routinely interact with conversational Artificial Intelligence (AI) at an accelerating rate. According to a 2023 McKinsey survey of global executives, around 80% of respondents experimented with generative AI (1). AI interactions will likely become even more common as generative AIs increasingly serve in roles previously reserved for humans such as managers (2), tutors (3), negotiators (4), and even engaging in political discourse (5). Furthermore, in many contexts, people now seamlessly alternate between interacting with AIs and humans, such as when speaking with customer service chatbots before being directed to human representatives, or when chatbots appear side-by-side with humans on popular messaging platforms (6).

Existing work has focused on nonconversational AI, such as systems used in medical diagnoses or weather predictions (7–9). However, people are increasingly working with conversational AI on tasks formerly reserved for humans, such as those that require creativity (10). Our work builds on prior research by offering insight into people's interactions with conversational AI agents collaborating on such tasks and uniquely informs how these interactions impact their later evaluations of other people.

**Fig. 1.**
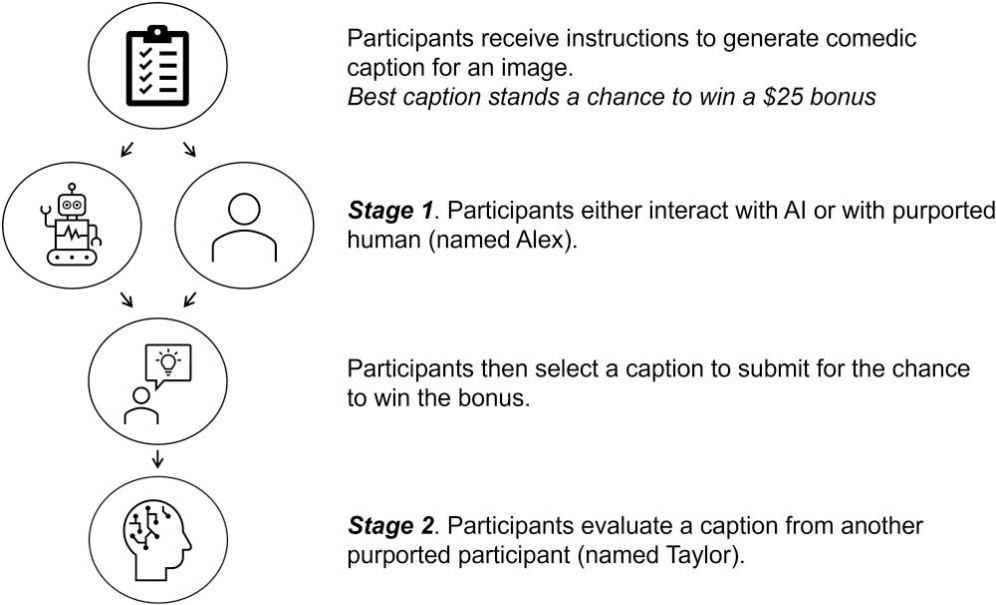
Step-by-step procedure for our experimental paradigm.

Our research asks two fundamental questions: First, how do people communicate with AIs compared with humans? Second, does interacting with AI in turn shape how people judge other humans? In two preregistered experiments (Total *N* = 1,261), participants were randomly assigned to work on a financially consequential task in collaboration with either an AI or another purported human participant (see Fig. 1). Specifically, they worked with their partner on coming up with a funny image caption. Unbeknownst to participants, the interaction partner in both conditions was GPT-4, with the purported human's prompt tweaked to resemble human responses in that condition (see Supplementary Material, Appendix). This design allowed us to isolate the impact of participants’ perceptions of their interaction partners while controlling for other factors by which humans and AI agents differ (e.g. AI agents’ vast knowledge base).

Then, to assess whether the initial interaction shaped people's subsequent evaluations of others’ work, participants rated yet another purported participant's caption. Lastly, participants were debriefed.

All studies were approved by the University of Pennsylvania's Institutional Review Board (IRB). Informed consent was obtained from all participants prior to their participation.

## Results

### Stage 1 natural language processing analyses

**Fig. 2.**
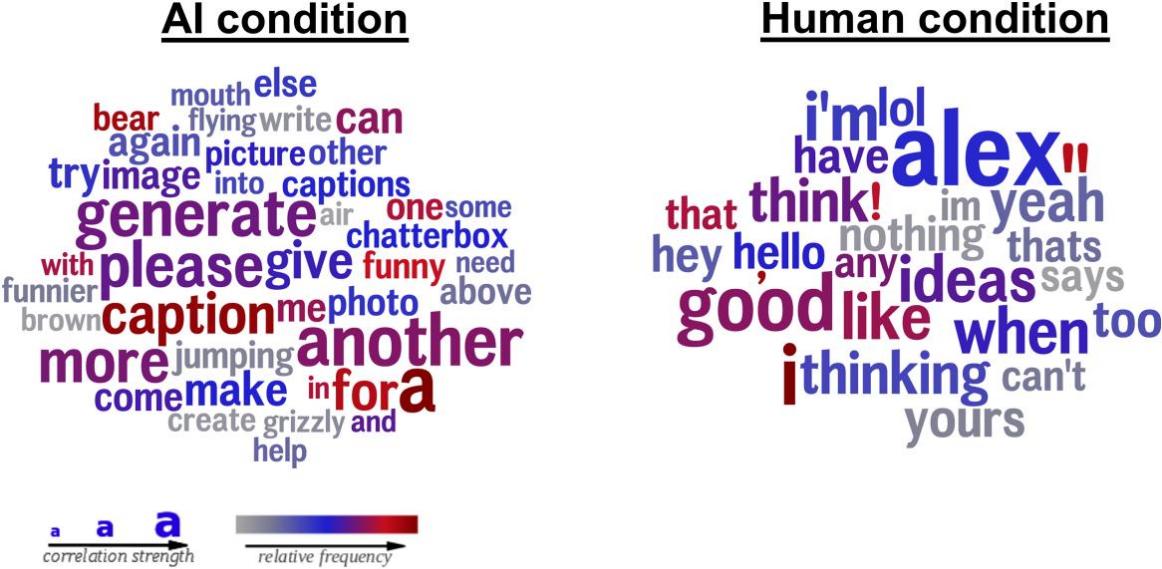
Word clouds representing the words and phrases most strongly associated with talking to AIs and humans. Words were extracted using DLATK using data from both studies. Color represents word frequency, with gray-red representing infrequent-frequent. Size represents the strength of the association between a given word and its associated condition, with larger words being more strongly tied to their respective condition. “Alex” in the human condition represents the name of the purported interaction partner in the human condition.

Given that both studies were identical in Stage 1, we pooled the data across the two studies in our analysis. In exploratory analyses, we first used the Differential Language Analysis ToolKit (DLATK; 11) to extract the words and phrases most closely associated with each experimental condition from participants’ input (see Fig. 2). We then used GPT-4 to code the same participants' interactions with their partners on various dimensions (in a sensitivity analysis, we also replicated the same coding pattern using human coders, see Supplementary Material, Appendix; 12).

**Fig. 3.**
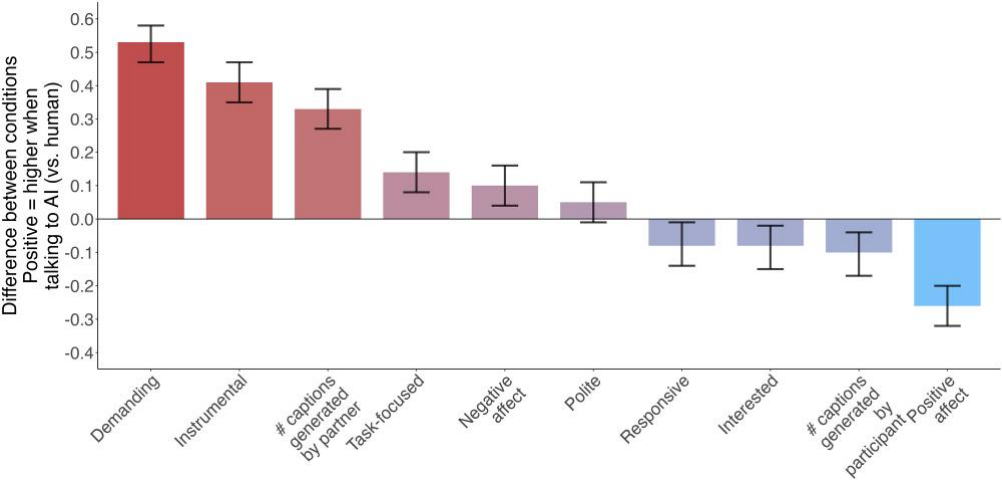
Standardized effect sizes comparing AI and human conditions. The bars represent differences between conditions in conversational features. Error bars represent 95% CIs.

Using linear regressions, we examined the effect of condition (AI versus Human) and a study indicator on each GPT-annotated dimension, with *P*-values adjusted for multiple comparisons using Benjamini & Hochberg's procedure (see Fig. 3 and Table S1 in Supplementary Material, Appendix; 13). Participants in the AI (versus Human) condition treated their interaction partner with more demandingness, *β* = 0.53, *P* < 0.001, instrumentality, *β* = 0.41, *P* < 0.001, less positive affect, *β* = −0.26, *P* < 0.001, more negative affect, *β* = 0.10, *P* = 0.003, higher task engagement, *β* = 0.14, *P* < 0.001, less responsiveness, *β* = −0.08, *P* = 0.017, and lower interest in their partner, *β* = −0.08, *P* = 0.011. AI condition participants also had their partner generate more captions, *β* = 0.33, *P* < 0.001, and generated fewer captions themselves, *β* = −0.1, *P* = 0.002. The conditions did not significantly differ on politeness, *β* = 0.05, *P* = 0.112.

### Stage 2 caption ratings

**Fig. 4.**
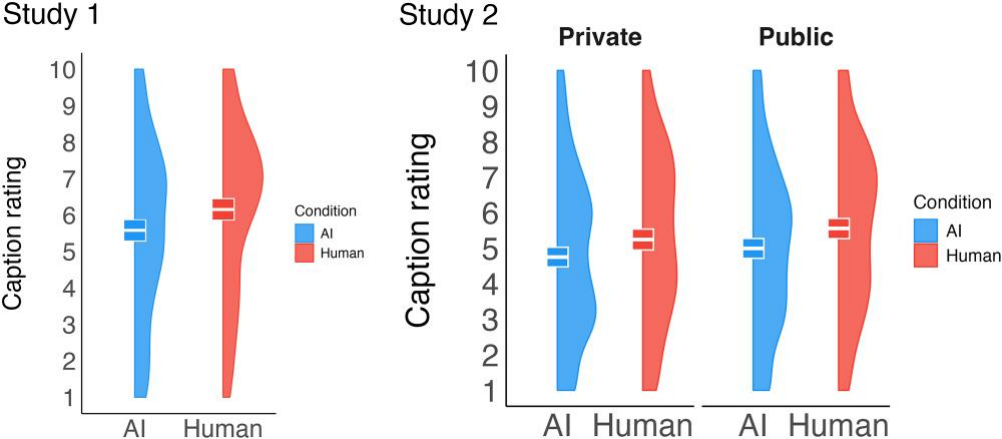
Stage 2 caption ratings by condition. White horizontal bars represent the mean, and colored boxes represent 95% CIs.

We examined the spillover effect of participants’ first interaction on the subsequent evaluation of an unrelated caption using Welch's two-sample *t*-tests. In Study 1, participants in the AI condition rated the subsequent participant's caption significantly lower than participants in the Human condition *t*(496.72) = 2.74, *P* = 0.003, *d* = 0.24 (see Fig. 4). We replicate this finding in Study 2, where we additionally examine whether the effect depends on whether participants are told that the participant whose caption they are judging will (public measure) or will not (private measure) be made aware of their caption's evaluation. Participants in the AI (versus Human) condition provided lower ratings on both the public, *t*(520.76) = 2.76, *P* = 0.006, *d* = 0.24 and private measure, *t*(518.51) = 2.40, *P* = 0.017, *d* = 0.21. The effect is not moderated by experience with conversational AI agents (see Supplementary Material, Appendix Section 6).

### Caption quality

A separate sample of participants (*N* = 946) judged how funny they found four randomly assigned captions from Studies 1 and 2. Ratings did not differ across the AI and Human conditions, *t*(2,828) = −0.18, *P* = 0.86. Thus, the effect does not seem to be driven by participants’ seeing better captions across conditions.

## Discussion

In two preregistered studies, participants who worked with AI (versus a purported human) were more demanding and instrumental, while displaying less positive affect. Importantly, this demanding attitude spilled over to another unrelated task, negatively coloring how participants judged another purported participant. This pattern arose despite the initial interactions being brief (under 3 minutes), and even though the interactions were completely unrelated to the subsequent evaluation task.

This negative spillover highlights a harmful downstream consequence of the rise in human–AI interactions. That is, people's interaction style when talking with AI may spread to their subsequent evaluations of humans, with potentially harmful consequences on interpersonal relationships writ large.

Notably, participants’ attitudes toward the AI and the subsequent spillover emerged despite the fact that participants in both conditions interacted with the same AI model with the same knowledge base. This suggests that the effect was rooted in participants’ perceptions of AI (versus humans), rather than any actual differences in capabilities.

In our research, participants evaluated another person immediately after interacting with an AI (or purported human). An important consideration for future work is the extent to which this spillover persists over time. Knowing this effect's temporal course could inform interventions to address it, such as a “cool down” period between AI and important subsequent human evaluations.

Broadly speaking, our results represent a practical drawback to the mass adoption of AIs in personal and professional task collaborations. We believe understanding how people collaborate with AIs represents an exciting and urgent area of inquiry.

## Materials and methods

All studies were approved by the University of Pennsylvania's IRB (#853653). Both preregistered experiments employ the same experimental paradigm (see Fig. 1). Prolific Academic participants (*N*_Study 1_ = 500; *N*_Study 2_ = 761) were recruited and randomly assigned to generate a caption for a comedic photo in collaboration with either an AI (AI condition) or a purported human, “Alex” (Human condition). In fact, all participants interacted with GPT-4 (with slight differences in prompts that map more closely to AI or human speech; see Supplementary Material, Appendix for prompts). Participants were also told that the 10 best captions stood a chance to win a $25 bonus.

After participants interacted with GPT-4 to generate comedic captions for 2.5 minutes, they rated another caption made by “Taylor,” a purported other participant (“Im bearly full!” [*sic*]; *1* = “Very poor,” *10* = “Exceptional”) and explained that rating to the purported participant (Study 1). In Study 2, participants rated the same caption twice, once for the researchers to receive the rating and another for Taylor to view, without explaining the rating. Importantly, the two conditions did not differ on an exploratory measure tapping participants’ suspicion that the participant whose caption they rated was a bot (*1* = “definitely human”, *5* = “definitely bot”), *P* = 0.918.

Per our preregistration, only participants who had interacted with AI more than once or twice before were included (see Section 1 of Supplementary Material, Appendix for details). In addition, participants in Study 2 were only included in the analyses if they passed a comprehension check (*N* = 526 of 761, 69%). Nonetheless, qualitatively identical results emerge when including all participants in the analyses (see Section 2 of Supplementary Material, Appendix).

## Supplementary Material

pgae397_Supplementary_Data

## Data Availability

Preregistration, materials, data, and analytic code for both studies are available at https://osf.io/fy6en/? view_only=c15144a7d58a41929573b777b4b4b94e.
