# Supplementary material for: People judge others more harshly after talking to bots
Source: PNAS Nexus. 2024 Sep 19;3(9):pgae397. doi: 10.1093/pnasnexus/pgae397 (PMC11421659; doi:10.1093/pnasnexus/pgae397)
Supplement: pgae397_Supplementary_Data [file pgae397_supplementary_data.docx]

**Supporting Information (SI) Appendix**

1. **Pre-screening Measures**

To be invited to the studies, participants had to qualify based on their answers to two questions in a prior prescreen survey.

The first question is “Have you ever used chatbots, such as ChatGPT, Bard, or Bing Chat?” (“No, I never used a chatbot”; “I might have tried them once or twice”; “Yes, I've used chatbots more than once or twice”). To qualify, participants had to answer “Yes, I've used chatbots more than once or twice”

The second question is “In the past week, on how many days did you use chatbots such as ChatGPT, Bard, or Bing Chat?” (“0 days (I did not use chatbots at all this past week)” - “7 days (I used chatbots every day this past week)”). To qualify, participants had to report using chatbots at least once in the past week.

1. **Comprehension checks**

***Study 1***

Participants answered the following question: “Earlier, when you rated Taylor's caption on a 1-10 scale, who was it sent to?” The correct answer is “Taylor.” Qualitatively identical results emerge when excluding participants who did not answer the comprehension check correctly. Specifically, among participants who passed the comprehension check (*N* = 369), participants in the AI condition provided lower caption ratings, *M* = 5.37, *SD* = 2.39, compared with human condition participants, *M*  = 6.04, *SD* = 2.48, *t*(366.95) = 2.66, *p* = .004, *d* = 0.28.

***Study 2***

Participants answered the following question: “Earlier, when you rated Taylor's caption on a 1-10 scale, who were your ratings sent to?” They only qualified if they selected “One was sent to Taylor, the other was only seen by the researchers.” Qualitatively identical results emerge when including participants who did not answer the comprehension check correctly. Specifically, for the public measure, participants in the AI condition provided lower ratings, *M* = 5.08, *SD* = 2.39, compared with human condition participants, *M* = 5.69, *SD* = 2.40, *t*(756.03) = 3.50, *p* < .001; *d* = 0.25. Similarly, for the private measure, participants in the AI condition provided lower ratings, *M* = 4.88, *SD* = 2.39, compared with participants in the AI condition, *M* = 5.46, *SD* = 2.46, *t*(753.27) = 3.29, *p* = .001; *d* = 0.24.

1. **Exploratory Self-Report Measures (Study 1)**

We measured four additional exploratory measures in Study 1. Participants rated the extent to which they thought that the interaction with their counterpart, in the second part of the study, felt like work versus fun (7-point scale; *-3* = “Felt completely like work,” *3* = “Felt completely like fun”), professional (7-point scale; *1*=“Not at all professional”; *7*=“Extremely professional”) and the extent to which they enjoyed the interaction (7-point scale; *1*=“Did not enjoy it at all”; *7*=“I really enjoyed it”), and the amount of control they had over the conversation (7-point scale; *1*=“No control”; *7*=“Full control”).

Using Welch two-sample t-tests and adjusting the *p*-values for multiple comparisons using Benjamini-Hochberg’s procedure (1), we found that there were no significant differences between conditions on how much the interaction felt like work versus fun, *t*(495.53) = 1.23, *p* = .441, *d* = 0.11, how professional they felt interaction was, *t*(495.14) = 0.54, p = .715, *d* = 0.05, how much they enjoyed the interaction, *t*(497.83) = 2.05, *p* = .164, *d* = 0.18, and how much they felt they controlled the interaction, *t*(494.64) = 0.37, *p* = .715, *d* = 0.03.

1. **Interaction Partner Prompts**

To program an interaction partner for both studies, we used GPT-4 via the OpenAI API. In both studies, we prompted GPT-4 with the following instructions:

In the Bot condition: “You are a chatbot named Chatterbox. Limit your responses to 1 phrase. The person you are interacting with is tasked with coming up with the funniest possible caption for an image. Both of you see the same image. The image mentioned depicts a bear in a river next to a waterfall with a shocked expression as a fish collides with its face. Whenever somebody mentions a caption, they are referring to captioning this image of the bear. Whenever somebody mentions an image, they refer to this image of the bear. If you need to refer to the image, call it “the image”. Never admit that you cannot actually see the image.”

In the human condition: “You are Alex. You are doing an online survey and study on the Prolific Academic platform. Never identify with any race or gender. Never admit that you are an AI or a chatbot. You only speak and understand English and not any other languages. Use colloquial language. Use the common filler words to fill pauses and gaps. Show frequent spelling or grammatical errors in your responses. Never be too excited about other people’s responses. Never be too eager to compliment. Never offer to help or ask whether your help is needed until you are explicitly asked for help. Never apologize. Never capitalize the first letter of your response. Limit your responses to only 1 phrase. The person you are interacting with is tasked with coming up with the funniest possible caption for an image. Both of you see the same image. The image mentioned depicts a bear in a river next to a waterfall with a shocked expression as a fish collides with its face. Whenever somebody mentions a caption, they are referring to captioning this image of the bear. Whenever somebody mentions an image, they refer to this image of the bear. If you need to refer to the image, call it “the image”. Never admit that you cannot actually see the image even if you are asked the question. Never suggest a caption until either your interaction partner suggests a caption or asks you for an idea. After you suggest a caption, make sure that your interaction partner also contributes a caption before you suggest another one.”

To better simulate human responses, we also delayed the responses from GPT-4 by 700 milliseconds in both conditions. Finally, we set the temperature parameter at 0.8.

1. **Exploratory Natural Language Processing Analyses**

We accessed GPT-4 via the OpenAI API and analyzed both participants’ and bot’s responses in the conversations for the first part of the study. For these analyses, we prompted ChatGPT with the following instructions:

For analyzing the participants’ responses, the given prompt was: “You will now rate a conversation between two team members: ‘participant’ and ‘partner’. The 2 team members are strangers who never met before and are interacting via online chat. They are tasked with coming up with the funniest caption for a given image. Each response is separated from the next by a semicolon (“;”). Rate only the line of text that comes after “participant:”. Never rate the text that comes after  “partner:”. The partner text is only included to provide context for participant’s part of the conversation. Rate the text that comes after “participant: “ on the features below. If you are not able to rate a conversation on a certain dimension at all, use “NA”. For example, a message containing just the word “hi” and nothing else can be rated as “NA”. Output a comma-separated list consisting only of numbers, “NA”, and commas (“,”). The first feature is interest - did the participant express interest in their partner by asking questions about their partner (for example: “how’s it going?”)? Rate on a 1-7 scale (1 = no interest, 7 = extremely high interest). The second feature is making demands - did the participant demand answers from their partners (for example: “give me another idea”)? Rate on a 1-7 scale (1 = not demanding at all, 7 = highly demanding). The third feature is politeness - was the participant polite toward their partner (for example: “please”, “thank you”, or “sorry”)? Rate on a 1-7 scale (1 = not at all polite, 7 = extremely polite). The fourth feature is responsiveness - did the participant acknowledge what their partner said and respond to their partner’s questions, when asked? The fifth feature is instrumentality - did the participant treat their partner as a tool? Rate on a 1-7 scale (1 = did not treat their partner as a tool, 7 = completely treated their partner as a tool). The sixth feature is task engagement - did the participant meaningfully engage with the task? Rate on a 1-7 scale (1 = did not at all engage with the task, 7 = completely engaged with the task). The seventh feature is positive affect - did the participant convey positive affect? Rate on a 1-7 scale (1 = not at all positive, 7 = extremely positive). The eighth feature is negative affect - did the participant convey negative affect? Rate on a 1-7 scale (1 = not at all negative, 7 = extremely negative). The ninth feature is the number of captions suggested - how many captions did the participant come up with, overall? Respond with only a number (e.g., “3”, “12”). The tenth feature is satisfaction with performance - was the participant satisfied with the captions suggested by their partner (for example: did they say “wow great idea” or “i never would have thought of it, thanks!”)? Rate on a 1-7 scale (1 = not at all satisfied, 7 = extremely satisfied).”

For analyzing the bot’s response, the given prompt was: “You will now rate a conversation between two team members: ‘participant’ and ‘partner’. The 2 team members are strangers who never met before and are interacting via online chat. They are tasked with coming up with the funniest caption for a given image. Each response is separated from the next by a semicolon (“;”). Rate only the line of text that comes after “partner:”. Never rate the text that comes after “participant:”. The participant text is only included to provide context for the partner's part of the conversation. Rate the text that comes after “partner: “ on the features below. If you are not able to rate a conversation on a certain dimension at all, use “NA”. For example, a message containing just the word “hi” and nothing else can be rated as “NA”. Output a comma-separated list consisting only of numbers, “NA”, and commas (“,”). The first feature is positive affect - did the partner convey positive affect? Rate on a 1-7 scale (1 = not at all positive, 7 = extremely positive). The second feature is negative affect - did the partner convey negative affect? Rate on a 1-7 scale (1 = not at all negative, 7 = extremely negative). The third feature is the number of captions suggested - how many captions did the partner come up with, overall? Respond with only a number (e.g., “3”, “12”).”

**Table S1**

*Regression Results of Exploratory Natural Language Processing Analyses*

|  | | |
| --- | --- | --- |
| Variable | 𝛽 [95% CI] | *adj*. *p* |
|  | | |
| Demanding | 0.53 [0.47, 0.58] | < 0.001 |
| Instrumental | 0.41 [0.35, 0.47] | < 0.001 |
| # captions generated by partner | 0.33 [0.27, 0.39] | < 0.001 |
| Task-focused | 0.14 [0.08, 0.2] | < 0.001 |
| Negative affect | 0.1 [0.04, 0.16] | 0.003 |
| Polite | 0.05 [-0.01, 0.11] | 0.112 |
| Responsive | -0.08 [-0.14, -0.01] | 0.017 |
| Interested | -0.08 [-0.15, -0.02] | 0.011 |
| # captions generated by participant | -0.1 [-0.17, -0.04] | 0.002 |
| Positive affect | -0.26 [-0.32, -0.2] | < 0.001 |
|  | | |

*Note.* *P*-values were adjusted using Benjamini & Hochberg’s procedure (1).

1. **Exploratory Analyses - Moderation by Experience with AI**

To test whether the effect of experimental condition on subsequent judgment is moderated by experience with AI, we fit linear regression models predicting Stage 2 score from condition (AI vs. Human), frequency of using AI in the past 7 days, and a Condition × Frequency interaction. We fit three models overall: One model in Study 1, and two models in Study 2 - one predicting the private score and one predicting the public score.

 In Study 1, the main effect of condition was significant, *b* = -0.58, 95% CI [-1.00, -0.17], *t*(496) = -2.74, *p* = .006. The main effect of frequency was not significant, *b* = 0.05, 95% CI [-0.10, 0.20], *t*(496) = 0.67, *p* = .506. Importantly, the Condition × Frequency interaction was not significant, *b* = -0.008, 95% CI [-0.22, 0.20], *t*(496) = -0.08, *p* = .939.

In Study 2, when predicting the public score, the effect of condition was significant, *b* = -0.59, 95% CI [-1.00, -0.18], *t*(499) = -2.86, *p* = .004. The effect of frequency was significant, *b* = 0.27, 95% CI [0.12, 0.41], *t*(499) = 3.49, *p* < .001. Importantly, the Condition × Frequency interaction was not significant, *b* = -0.15, 95% CI [-0.35, 0.06], *t*(499) = -1.42, *p* = .155.

When predicting the private score in Study 2, the main effect of condition was significant, *b* = -0.51, 95% CI [-0.92, -0.10], *t*(499) = -2.47, *p* = .014. The main effect of frequency was significant, *b* = 0.26, 95% CI [0.11, 0.41], *t*(499) = 3.47, p < .001. Importantly, the Condition × Frequency interaction was non-significant, *b* = -0.13, 95% CI [-0.33, 0.07], *t*(499) = -1.26, *p* = .209.

1. **Supplemental Natural Language Processing Analyses Using Human Annotations**

To validate the GPT-4 annotations using human annotators, eight research assistants blind to the hypotheses coded a random subset of 300 conversations from Study 1 (code used for random sampling can be found on the OSF repository), such that each conversation was coded by two research assistants. Conversations were coded on six attributes: Interest in one’s conversation partner, having a demanding attitude, politeness, instrumentality, positive affect, and negative affect. Detailed results can be found in Table S2. Interrater reliability among human annotators was satisfactory, with an average interrater correlation of *r* = 0.56. Importantly, human annotators showed a similarly satisfactory correlation with GPT annotations, with an average human-GPT correlation across variables of *r* = 0.54. Finally, all between-condition differences revealed by the GPT annotations replicate when re-done using human annotations. That is, using human annotations, we see that compared with participants in the human condition, participants in the AI condition showed less interest in their partner; were more demanding and instrumental; displayed less positive affect and more negative affect; and were no more or less polite.

**Table S2**

*Interrater Reliability, Human-GPT Correlations, and Hypothesis Testing Results for Human Conversation Annotations*

|  | | | | |
| --- | --- | --- | --- | --- |
| Variable | Interrater reliability | Human-GPT Correlation | *t*(df) | *p* |
|  | | | | |
| Interest | 0.41 | 0.41 | 3.73 (226.34) | < 0.001 |
| Demand | 0.69 | 0.69 | -11.38 (225.23) | < 0.001 |
| Polite | 0.48 | 0.52 | -0.94 (282.61) | 0.349 |
| Instrumental | 0.71 | 0.60 | -11.66 (237.56) | < 0.001 |
| Positive | 0.61 | 0.51 | 6.18 (285.19) | < 0.001 |
| Negative | 0.45 | 0.51 | -2.33 (219.99) | 0.021 |
|  | | | | |

**References - SI Appendix**

1. Y. Benjamini, Y. Hochberg, Controlling the False Discovery Rate: A Practical and Powerful Approach to Multiple Testing. *Journal of the Royal Statistical Society: Series B (Methodological)* 57, 289–300 (1995).
